# *LRP1B* mutation is associated with tumor HPV status and promotes poor disease outcomes with a higher mutation count in HPV-related cervical carcinoma and head & neck squamous cell carcinoma

**DOI:** 10.7150/ijbs.56970

**Published:** 2021-04-22

**Authors:** Can-hui Cao, Rang Liu, Xin-ran Lin, Jia-qi Luo, Li-juan Cao, Qiu-ju Zhang, Shou-ren Lin, Lan Geng, Zhong-yi Sun, Si-kang Ye, Zhi-ying Yu, Yu Shi, Xi Xia

**Affiliations:** 1 Center for Reproductive Medicine, Department of Obstetrics and Gynecology, Peking University Shenzhen Hospital, Shenzhen Peking University-The Hong Kong University of Science and Technology Medical Center, Guangdong, 518036, China.; 2 Department of Critical Care Medicine, The Eighth Affiliated Hospital, Sun Yat-sen University, Shenzhen, China.; 3 Department of Gynecology, The First Affiliated Hospital of Shenzhen University, Health Science Center, Shenzhen Second People's Hospital, Shenzhen, Guangdong, China.

**Keywords:** *LRP1B* mutation, tumor HPV status, cervical carcinoma, head and neck squamous cell carcinoma, tumor mutation count

## Abstract

Human papillomavirus (HPV) infection and gene mutations were reputed as key factors in cervical carcinoma (CC) and head and neck squamous cell carcinoma (HNSCC). However, the associations of HPV status and gene mutations remain to be determined. This study aims to identify molecular patterns of *LRP1B* mutation and HPV status via rewiring tumor samples of HNSCC (n=1478) and CC (n=178) from the TCGA dataset. Here, we found that *LRP1B* mutation was associated with HPV status in CC (*P*=0.040) and HNSCC (*P*=0.044), especially in HPV 16 integrated CC (*P*=0.036). Cancer survival analysis demonstrated that samples with *LRP1B* mutation showed poor disease outcomes in CC (*P*=0.013) and HNSCC (*P*=0.0124). In addition, the expression status of* LPR1B* was more favorable for prediction than *TP53* or *RB1* in CC and HNSCC. Mutation clustering analysis showed that samples with *LRP1B* mutation showed higher mutation count in CC (*P*=1.76e-67) and HNSCC (*P*<10e-10). Further analysis identified 289 co-occurrence genes in these two cancer types, which were enriched in PI3K signaling, cell division process, and chromosome segregation process, et al. The 289-co-occurrence gene signature identified a cluster of patients with a higher portion of copy number variation (CNV) lost in the genome, different tumor HPV status (*P*<10e-10), higher mutation count (*P*<10e-10), higher fraction genome altered value (*P*=2.078e-4), higher aneuploidy score (*P*=3.362e-4), and earlier started the smoking year (*P*=2.572e-4), which were associated with shorter overall survival (*P*=0.0103) in CC and HNSCC samples. Overall, *LRP1B* mutation was associated with tumor HPV status and was an unfavorable prognostic biomarker for CC and HNSCC.

## Introduction

Human Papillomavirus (HPV) infection was reported to be responsible for almost all cervical cancer (CC), more than 90% of anal cancer, almost 70% of vaginal and vulvar cancers, about 60% of penile cancer, 5% to 30% of head and neck squamous cell carcinoma (HNSCC), and 60% to 70% of oropharynx cancer 1-4. According to the statistics of the Centers for Disease Control (CDC), about 3% of all cancers in women and 2% in men are caused by high-risk HPVs, with 34,000 new cases per year. Worldwide, about 4.5% of all cancers are caused by high-risk HPVs, with 630,000 (570,000 women and 60,000 men) new cases each year 5.

In general, almost all sexually active people will be infected with at least one type of HPV, and around half of those infections are high-risk HPV types 6. Most HPV infections become negative after a few months and about 91% of them become undetectable after two years 7, but high-risk HPV infections persist longer time on average than low-risk HPV infections 8. Persistent high-risk HPV infections were thought to lead to genomic instability and local immune suppression, leading to genomic alteration accumulation and viral genome integration 9. It is increasingly appreciated that persistent high-risk HPV infections promote somatic mutation accumulation, which is responsible for the development of precancerous lesions or malignant carcinomas 10. The evolution distance of subclones in cancer is variable, even though cancer was reported to arise from the acquisition of multiple mutations that synergistically transform normal cells 11. Still, the associations between high-risk HPV infections and HPV-associated gene mutation remain to be determined.

Low-density lipoprotein receptor-related protein 1B (*LRP1B*) is reputed as a tumor suppressor gene on chromosome 2. Gene mutations in *LRP1B* are frequently detected in many cancers, with 12% in non-small cell lung cancer, 11% in head and neck cancer, 9% in cervical cancer, 8% in bladder cancer, and 8% in prostate cancer 12. A previous melanoma study reported that *LRP1B* mutation was enriched in responders (34%) to immune checkpoint inhibitors (ICIs) compared with non-responders (3%) 13, which suggested* LRP1B* mutation may be an independent predictor for the response to ICI. Previous studies have led to the notion that the diverse clinical outcomes of CC and HNSCC are likely a reflection of the genetic heterogeneity of the tumor subclones from the gene expression profiles and the intratumor metabolic features 14, 15. The identification of molecular patterns or biomarkers of CC and HNSCC that could not only predict the tumor biology but help to further classify HPV-related cancers. Patients with poor biological behavior could then be identified and treated with individual therapies 16. In particular, *LRP1B* mutation was identified as a biomarker for ICIs in melanoma, non-small cell lung cancer 17, prostate cancer 18, and advanced biliary tract cancer 19.

Here, we correlated HPV status, somatic mutations, disease outcomes, HPV expression, mutation gene expression, and clinical information of CC and HNSCC samples from TCGA to systematically unravel the HPV-associated mutation gene, that is *LRP1B*. In addition, gene expression of *LRP1B* was used to calculate the receiver operating characteristic (ROC) curve between normal and tumor, which was synchronously compared to* TP53* or *RB1*. Furthermore, the co-occurrence mutation gene of *LRP1B* was performed to identify a co-occurrence mutation signature with different clinical characteristics in CC and HNSCC. These results suggested the associations of HPV status and *LRP1B* mutation, and the prognostic value of *LRP1B* in HPV-related CC and HNSCC.

## Material and Methods

### Cell culture

Cell resource and cell culture of SiHa and HeLa (human cervical cancer cell lines) were described previously 20. Cells were cultured in high glucose DMEM (12430054, Gibco) which contains 10% fetal bovine serum (SH30406.05, Hyclone) and penicillin-streptomycin (100 U/mL-100 μg/mL, 15140163, Gibco). Cells used were grown at 37 °C with 5% CO_2_.

### Immunofluorescence

According to the Catalogue of Somatic Mutations in Cancer (COSMIC) dataset, SiHa and MS751 were *LRP1B* wild type, Hela, Caski, and Me180 were *LRP1B* mutation cell lines (Supplementary Tables 1-3). Cells (1×10^4^) were seeded in glass coverslips in 24-well plates after 24 h and were fixed in 4% paraformaldehyde (30 min) at room temperature, then penetrated in 0.3% Triton X-100 (30 min) before the blocking step (5% Bovine serum albumin (B2064, Sigma-Aldrich) for 30 min in 37 °C) 21. Antibodies used for incubation were *LRP1B* (SAB4200326, Sigma-Aldrich, 1:200) and Alexa Fluor 594 (ANT029, Antgene, 1:100). Phalloidin (A12379, Invitrogen) and DAPI (C1002, Beyotime) were used to stain microfilaments and the nucleus. Images were taken using a microscope (BX53, Olympus) equipped with FITC and Cy3 filters. The mean intensity of staining was calculated by ImageJ (1.48v).

### Sample data acquisition

Integrated genomic and molecular data of 178 cervical carcinoma samples (169 HPV positive samples, 120 HPV-A9 positive samples, 103 HPV16 positive samples, and 78 HPV16 integration samples, Supplementary Table 4) were described as previously published as TCGA cohort 1 22, including HPV status of the tumor, somatic (point mutation) mutation information of samples, days-to-last-follow-up information of samples, E6 normalized counts (status: spliced or un-spliced). HNSCC samples were downloaded and performed from HNSCC cohorts of cBioPortal tool (https://www.cbioportal.org/) with default settings 4, 23-26. 21.9% of samples were with HPV status, 7.8% were with p16 status, 4.9% were with tumor HPV PCR, and 4.4% were with HPV *in situ* hybridization (ISH) status. Different research used different detection methods. 12.9% of samples with high-risk HPV were detected in RNA-Seq analysis 4; 14% of all samples and 53% of oropharyngeal tumors were found positive for HPV based on PCR analysis 23; 12.5% of samples showed HPV positive by ISH 24. Gene expression profiles of *LRP1B* and corresponding clinical information were downloaded from the Gene Expression Omnibus (GEO), including GSE107591, GSE9750, GSE7803, and GSE63514.

### HPV types and HPV status annotation

According to the previous study 22, the HPV A9 subgroup includes HPV16, HPV31, HPV33, HPV35, HPV52, and HPV58 types. The HPV A7 subgroup includes HPV 16, HPV18, HPV39, HPV45, HPV59, HPV68, and HPV70 (Supplementary Table 4). HPV status of cervical carcinoma was described as previously published 22, including HPV A9 integration, HPV A9 non-integration, HPV 16 integration, and HPV 16 non-integration. Detailed statistics of CC was shown in Supplementary Table 4. HPV status of HNSCC performed in cBioPortal tool (https://www.cbioportal.org/) from HNSCC cohorts 4, 23-26 with default settings, corresponding *LRP1B* mutation status of samples was download from the dataset.

### E6 splices from RNA-Seq

HPV-E6 splices from RNA-seq were conducted by the previous piplines 22. Transcript types included transcripts with the unspliced sequence of E6, transcripts spliced at E6 splice donor site (loci 226 of HPV16, loci 233 for HPV18).

### HPV-*LRP1B* integration map

*LRP1B* was detected as an HPV integration hotspot in CC 27. We mapped HPV-*LRP1B* integrated samples in *LRP1B* gene with transcription regulation elements (DNAase cluster, Txn factor ChIP, DNase I hypersensitive sites, and NHEK (a normal epithelium cell line) chromatin state discovery and characterization (ChromHMM)). Samples were downloaded from 135 samples with over 3,667 integration sites. HPV-*LRP1B* integrated samples were indicated by arrows. *LRP1B* gene and regulation elements were downloaded from the UCSC browser (http://genome.ucsc.edu/).

### Immunohistochemistry (IHC) score of *LRP1B*

IHC score of LRP1B in cervical carcinoma (n = 97) and normal cervix (n = 7) were downloaded from the published dataset 27, with HPV integration status information, including HPV breakpoint sites, integrated HPV type, human genome breakpoint sites. Tumor samples were clustered into three groups, including normal cervix, samples with HPV integration in *LRP1B* (n = 6), samples with HPV integration in other genes (n = 91). All integration sites and scores were annotated with default settings.

### Disease survival analysis

The Kaplan-Meier method was used to assess the difference in the disease survival of patients. Overall survival analysis of cervical carcinoma samples was performed by Gehan-Breslow-Wilcoxon test according to the follow-up information from TCGA cohort 1 22, and samples were clustered into one group with* LRP1B* mutation and the other group with *LRP1B* wild type. Overall survival analysis of HNSCC was performed by cBioPortal (https://www.cbioportal.org/) via Log Rank test. Samples were clustered into one group with* LRP1B* mutation and the other group with *LRP1B* wild type. The Log Rank test gives greater weight to distant differences in outcome events, i.e., it is sensitive to distant differences, while the Wilcoxon test gives greater weight to near-term differences in outcome events.

### Receiver operating characteristic (ROC) curve analysis

ROC curve analysis was employed to demonstrate the sensitivity and specificity of genes by risk score. ROC curves of *LRP1B*, *TP53*, and *RB1* were performed by GraphPad Prism (version 6.02) from GSE107591, GSE9750, GSE7803, and GSE63514. GSE107591 was expression profiling of 23 normal and 24 HNSCC tissues. GSE9750 was 24 normal cervical epithelium and 33 primary cervical tumors. GSE7803 was 21 invasive cervical squamous cell carcinomas, 7 high grade squamous intraepithelial lesions (HSIL) and 10 normal squamous cervical epithelial samples. GSE7803 included 24 normal, 62 HSIL lesions, 40 CIN3 lesions, and 28 cancers.

### Co-occurrence mutation genes of *LRP1B*

Co-occurrence mutation genes of *LRP1B* were performed by cBioPortal (https://www.cbioportal.org/), the bioinformatic tool of TCGA, with default settings. Briefly, mutation genes of samples were performed in CC and HNSCC, respectively. Co-occurrence mutation genes and mutual exclusive genes were analyzed by Fisher's Exact Test 28, samples were divided into groups with *LRP1B* mutation or not. Description of 289 genes was showed in Supplementary Table 5.

### Molecular characteristics of co-occurrence mutation genes

Molecular characteristics of 289 co-occurrence mutation genes were performed in CC and HNSCC via cBioPortal (https://www.cbioportal.org/) with default settings, including clinical information, tumor type, tumor HPV status, patient smoking history, mutation count, CNV status, aneuploidy score, et al. P-value <0.05 was considered statistically significant. All significant results were showed in Supplementary Table 6.

### Enrichment terms analysis

The enrichment terms analysis was performed to demonstrate the biological processes (GO) and Kyoto Encyclopedia of Genes and Genome (KEGG) pathways of 289 co-occurrence mutation genes of *LRP1B* in Metascape 29 with default settings, an online gene annotation tool with multiple authoritative data sources. Gene symbols of 289 co-occurrence mutation genes of *LRP1B* were imported, then “Homo sapiens” was selected for further analysis to perform the role of the input genes in biological processes and KEGG pathways. The top 20 pathways and process enrichment were showed.

### Mutation count analysis of tumor samples

The cervical carcinoma samples of a TCGA cohort (n = 178) were downloaded 22 with WGS data. Samples were classified into one group with *LRP1B* mutation (n = 12) and the other group with *LRP1B* wild type (n = 166). Then, the top 255 mutation genes of all samples were displayed to analyze the mutation count of those two groups. Mutation count of CC (n = 605, TCGA-CC cohort 2), HNSCC (n = 1477) and pan-cancer studies (MSKCC) (n = 1676) were analyzed by clustering samples into *LRP1B* altered group and *LRP1B* unaltered group in cBioPortal tool (https://www.cbioportal.org/).

### Statistical analyses

Data were analyzed by GraphPad Prism (version 6.02, GraphPad Software, Inc.) and SPSS 25.0 (SPSS Inc., USA). Data were expressed as the mean ± SD (standard deviation). Data were analyzed by Student's t-test (E6 counts) one-way analysis of variance (ANOVA) test (IHC score), Gehan-Breslow-Wilcoxon test (Overall survival (OS) for CC), Log-rank test (OS for HNSCC), Chi-Square test (mutation count analysis, T (minimum) ≥5, N ≥40, Chi-square test; 1 ≤ T (minimum) ≤5, N ≥40, adjusted Chi-square test; T (minimum)<1 or N<40, Fisher's exact test), and Kruskal Wallis Test (mutation count analysis, aneuploidy score), followed by least significant difference (LSD) testing, detail information was showed in Supplementary Table 6. *P*-value <0.05 was considered statistically significant.

## Results

### *LRP1B* mutation was associated with HPV status in CC and HNSCC

**Figure 1:**
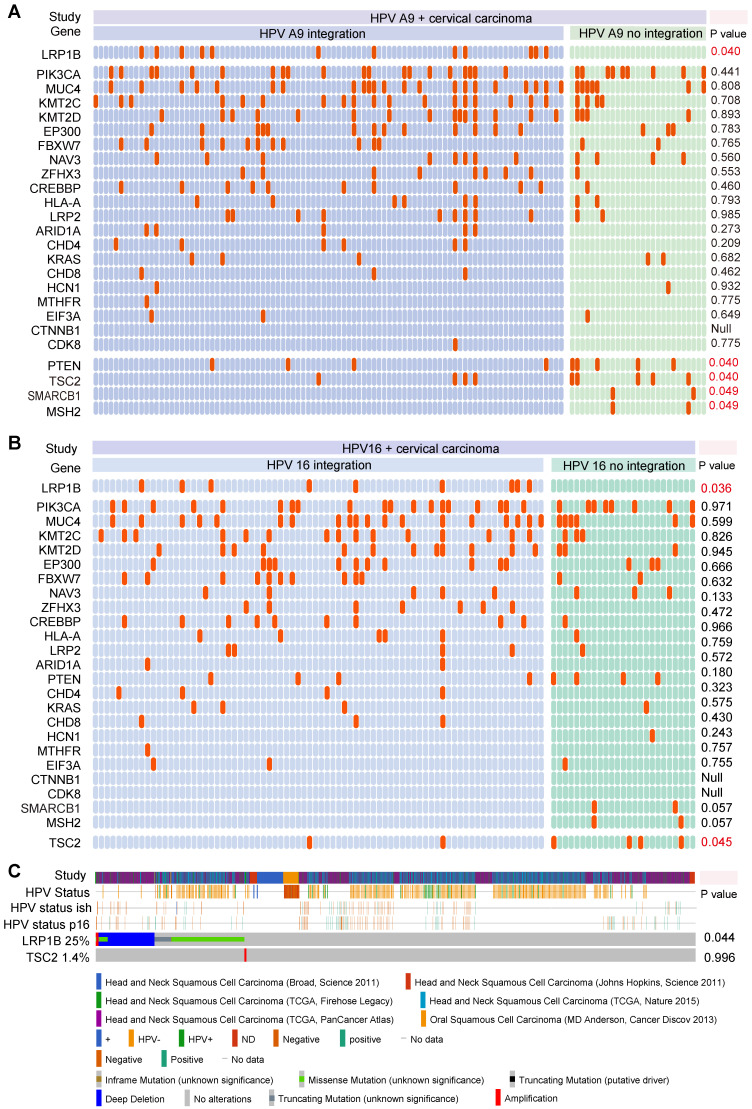
*** LRP1B* mutation was associated with HPV status in CC and HNSCC**. Mutation status and tumor HPV status in HPV A9 positive cervical carcinoma (**A**), in HPV 16 positive cervical carcinoma (**B**) and in head and neck squamous cell carcinoma (**C**). Each row should represent a gene while each column represents a sample, the orange column indicates mutation gene, top mutation genes in cervical samples are shown. P values were calculated by the Chi-square test.

To explore the associations between gene mutation and tumor HPV status, we integrated 169 CC samples from the TCGA cohort to investigate the associations between tumor HPV status and gene mutation in CC, we then performed correlation analysis in HPV-A9 positive (Figure 1A) and HPV-A7 positive (Figure S1) samples. We found that *LRP1B* mutation was associated with HPV-A9 integration status (*P* = 0.040), and PTEN mutation (*P* = 0.040), TSC2 mutation (*P* = 0.040), SMARCB1 mutation (*P* = 0.049), and MSH2 mutation (*P* = 0.049) were preferred in HPV-A9 non-integration samples.

HPV 16 (73.8%) infections are the most prevalent type in cervical cancer patients 30. We then performed the correlation analysis between HPV 16 status and gene mutation in the 103 samples (Figure 1B). *LRP1B* was found to be associated with HPV 16 integration status (*P* = 0.036) and TSC2 was preferred in HPV 16 non-integration group (*P* = 0.045). To further validate the correlation of *LRP1B* and TSC2 in HNSCC, we analyzed them in 1478 HNSCC samples and found that *LRP1B* was associated with tumor HPV status in HNSCC (P = 0.044, Figure 1C).

### HPV integration status was associated with increased E6 expression and reduced* LRP1B* expression

**Figure 2:**
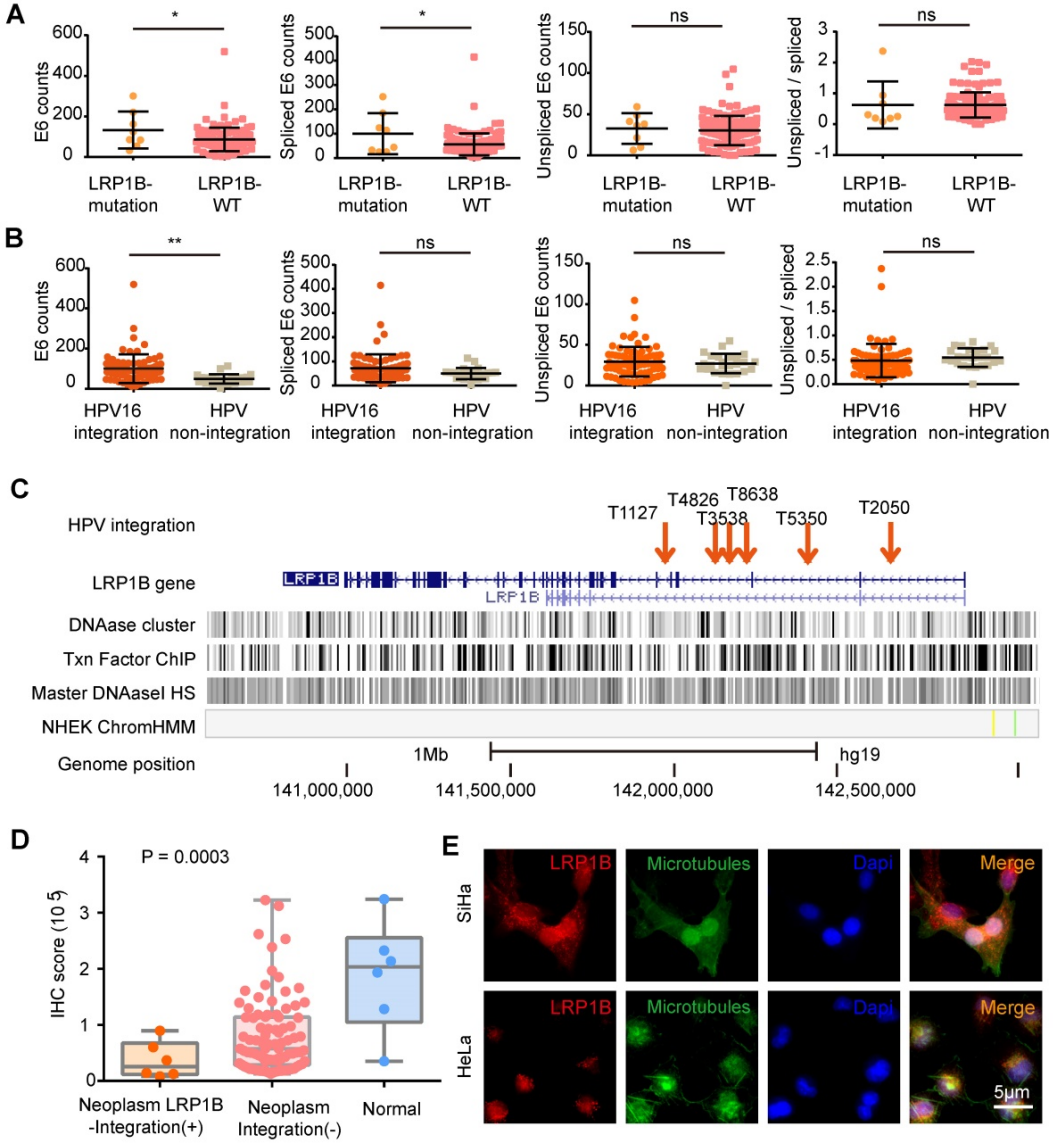
** HPV integration status was associated with increased E6 expression and reduced *LRP1B* expression.** (**A**) E6 (total/spliced/un-spliced) expression in *LRP1B* mutation samples and *LRP1B* wild type (WT) samples. E6 counts represented the numerical value from RNA-Seq data as following previous study 22. (**B**) E6 (total/spliced/un-spliced) expression in HPV 16 integration samples and HPV non-integration samples. (**C**) Distribution of HPV-*LRP1B* integration samples in a cervical carcinoma cohort 39. (**D**) IHC staining score of LRP1B in cervical carcinoma samples with different integration sites or normal samples from the same cohort 39. *P*-value was performed by One-way ANOVA. (**E**) Representative images of immunofluorescence staining of LRP1B in SiHa and Hela cells. Data are expressed as mean ± SD, * P < 0.05; ** P < 0.01).

Next, we assessed the effect of *LRP1B* mutation and E6 expression in CC (Figure 2A) and found that *LRP1B* mutation was associated with higher total E6 expression and spliced E6 expression, which was consistent with the E6 expression pattern in HPV 16 integration and non-integration samples (Figure 2B).

A previous study has reported *LRP1B* as an HPV integration hotspot in CC 27, with about 5.8% (6/104) frequency. By performing the HPV-*LRP1B* integration map in *LRP1B* genes (Figure 2C), we found that HPV was tended to integrate into the *LRP1B* gene region. Furthermore, when comparing samples with other HPV integration sites or normal samples, HPV-*LRP1B* integration was identified to decrease LRP1B expression in HPV-*LRP1B* integration samples (Figure 2D). HeLa cells with *LRP1B* mutation were found to show lower LRP1B staining than SiHa cells with *LRP1B* wild type (P=0.0048, Figure 2E, Figure S2A). Also, *LRP1B* expression of ME180 and Caski (*LRP1B* mutation) was lower than that of SiHa cells (P=0.0198, Figure S2B-D).

### *LRP1B* mutation was associated with poor disease outcome and *LRP1B* was identified as a prognostic biomarker in CC and HNSCC

**Figure 3:**
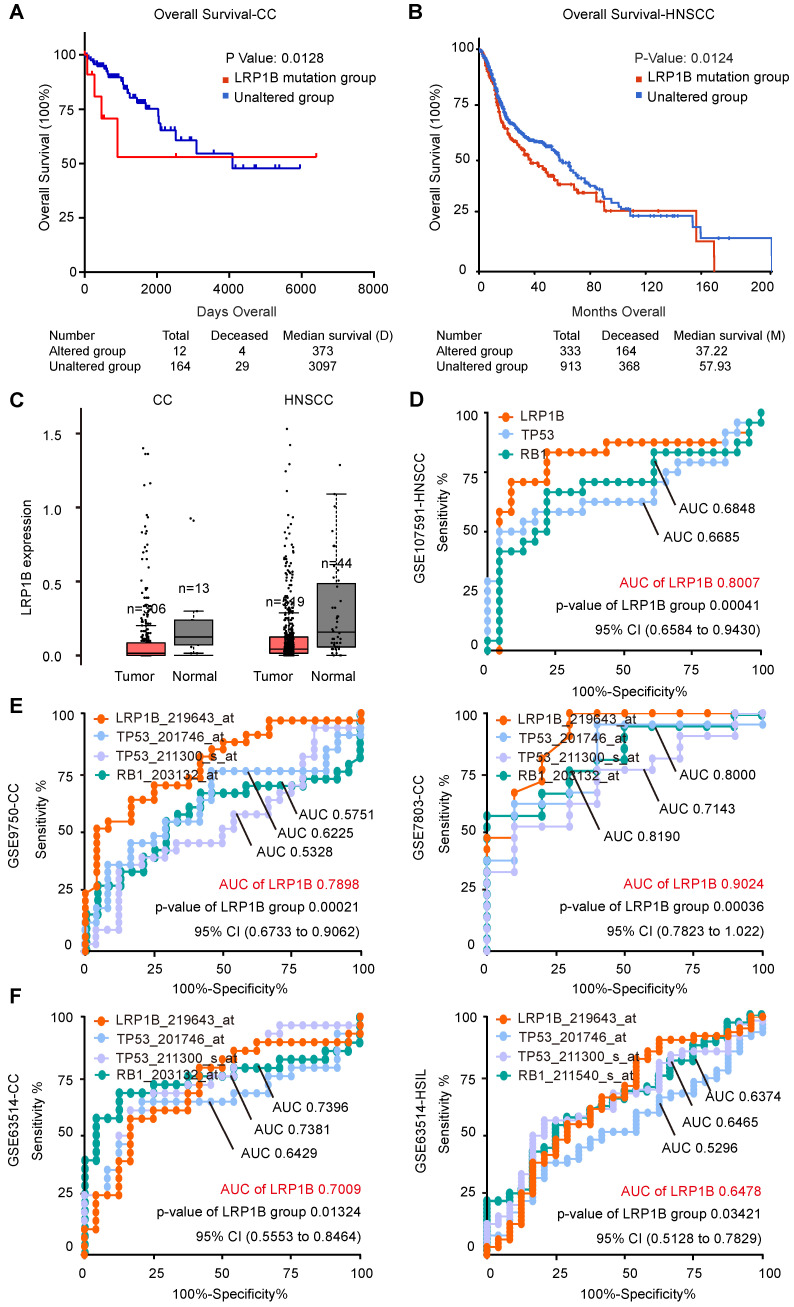
*** LRP1B* mutation was associated with poor disease outcomes and *LRP1B* was identified as a prognostic biomarker in CC and HNSCC.** Overall survival analysis of samples according to *LRP1B* mutation status in CC (**A**) and HNSCC (**B**). (**C**) *LRP1B* expression of tumor and normal in CC and HNSCC performed by GEPIA 40. ROC curve analysis of *LRP1B*, *TP53*, and *RB1* in GSE107591 (**D**), GSE9750, GSE7803 (**E**), and GSE63514 (**F**). CESE: cervical carcinoma, HNSCC: head and neck squamous cell carcinoma, HSIL: high grade squamous intraepithelial lesion.

In CC, we performed survival analysis in 178 samples with* LRP1B* mutation status (N (mutation) =14, N (wild type) = 164) and identified that *LRP1B* mutation was associated with poor disease outcome (*P* = 0.0135, Figure 3A). In HNSCC, we also found that* LRP1B* mutation (N = 333) was associated with poor disease outcome in 1478 samples (N (wild type) = 913, P = 0.0124, Figure 3B).

*LRP1B* expression between tumor and normal in CC and HNSCC showed no statistical differences (Figure 3C), and there was no significant difference between *LRP1B*-high expression and *LRP1B*-low expression group in CC and HNSCC (Figure S3). However, *LRP1B* expression was found to be a predictor in CC and HNSCC. ROC curve of *LRP1B*, *TP53*, and *RB1* was performed to analyze the prognostic value of *LRP1B* between normal and neoplasm samples. In HNSCC, *LRP1B* was found to show a higher ROC area than *TP53*, and *RB1* between normal and neoplasm samples (Figure 3D). In CC of GSE7803 and GSE9750, *LRP1B* was found to show a higher ROC area than TP53 and RB1 (Figure 3E). In GSE63514, *LRP1B* was found to show a similar ROC with TP53 and RB1 in CC and HSIL, with a slightly high trend (Figure 3F).

### *LRP1B* mutation was associated with a higher mutation count in CC and HNSCC

**Figure 4:**
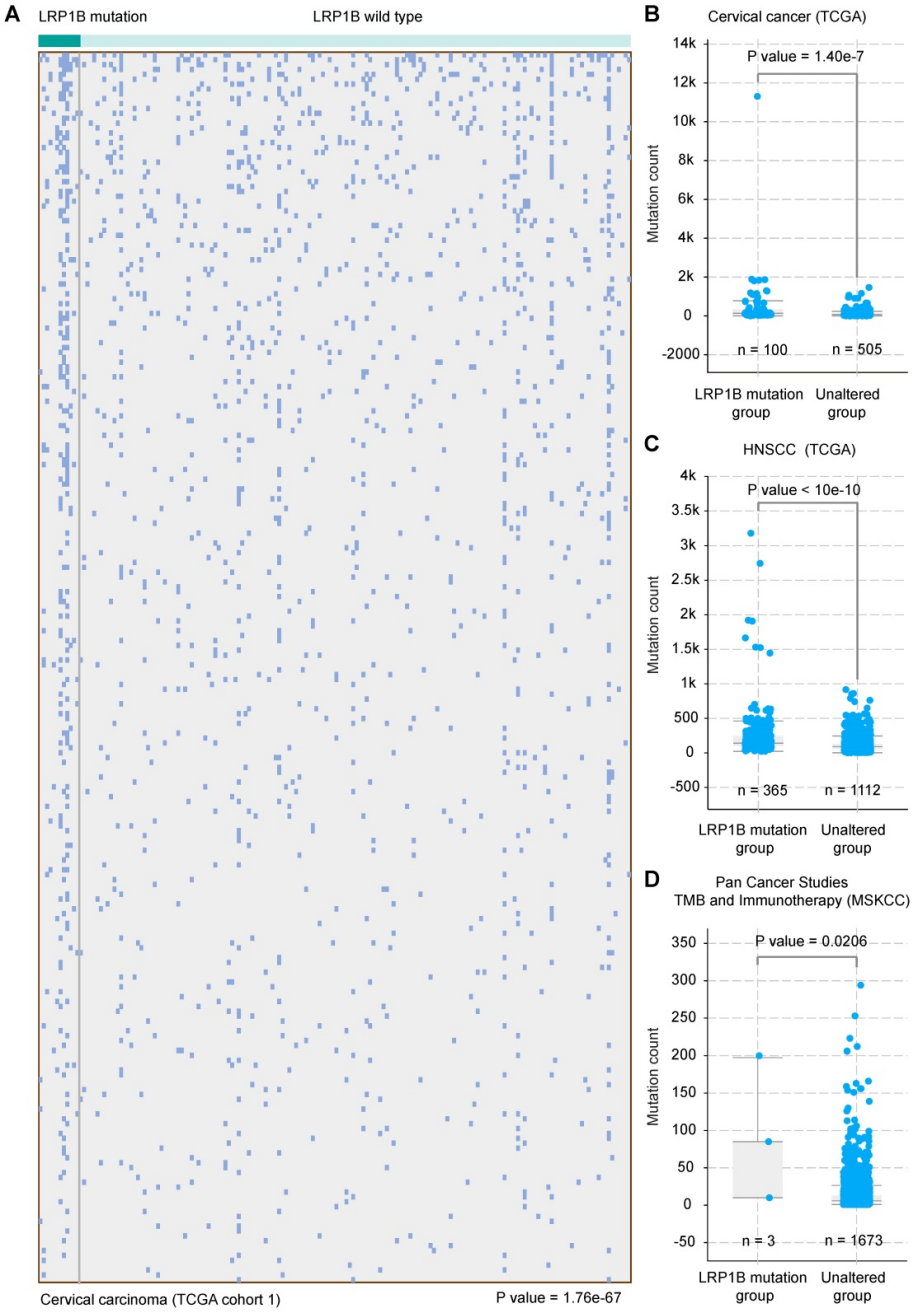
*** LRP1B* mutation was associated with a higher mutation count in CC and HNSCC.** (**A**) 178 cervical cancer samples (columns) were arranged by *LRP1B* mutation status, corresponding to the top 255 mutation genes (rows) of patients in cervical cancer. Each light blue column represents a somatic genomic alteration. P values were calculated by the Chi-square test. Mutation count analysis of samples according to *LRP1B* mutation status in TCGA-CC (**B**), in TCGA-HNSCC (**C**), and pan-cancer studies (**D**) from cBioPortal tool. P values were calculated by the Chi-square test.

CC and HNSCC were identified as the kind of tumor with high tumor mutational burden (TMB) and stable microsatellite instability (MSI) 31. Tumor mutational burden was reported as an independent biomarker for immunotherapy response in diverse cancers 32. To investigate the molecular basis of mutation count in samples with *LRP1B* mutation, we clustered the 178 CC samples according to *LRP1B* mutation status and found that *LRP1B* mutation status was associated with a higher mutation count in CC (*P* = 1.76e-67, Figure 4A), and in the combined TCGA-CC dataset (*P* = 1.40e-7, Figure 4B). In addition, *LRP1B* mutation status was identified to be associated with a higher mutation count in HNSCC (P < 10e-10, Figure 4C). Furthermore, *LRP1B* mutation status was also found to be associated with higher mutation count in pan-cancer studies (*P* = 0.0206, Figure 4D).

### Co-occurrence mutation signature of *LRP1B* in CC and HNSCC

**Figure 5:**
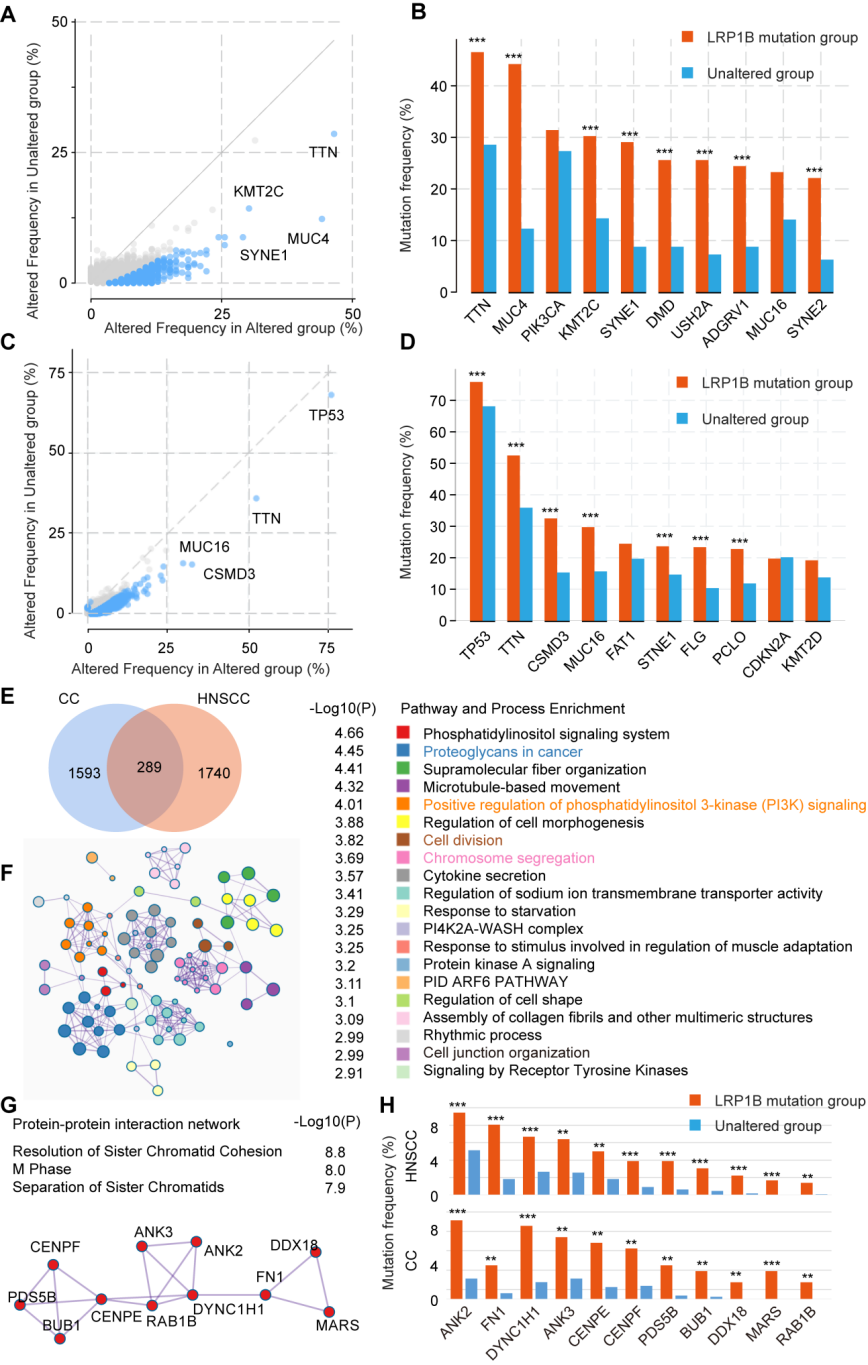
** Co-occurrence mutation signature of *LRP1B* in CC and HNSCC.** Co-occurrence mutation analysis of *LRP1B* and top 10 mutation genes of each group in CC (**A, B**) and in HNSCC (**C, D**) from cBioPortal tool. (**E**) Venn diagram of *LRP1B* co-occurrence mutation genes in CC and HNSCC. (**F**) Pathway and process enrichment analysis of 289 *LRP1B* co-occurrence mutation genes were performed by Metascape 29. (**G**) Protein-protein interaction network of chromosome segregation pathway. (**H**) Mutation frequency of genes relating the network in groups according to *LRP1B* mutation status. ** P < 0.01, *** P < 0.001.

To further explore the mutation characteristics of *LRP1B* mutation, we performed co-occurrence mutation analysis in CC and HNSCC, respectively. Several co-occurrence mutation genes were identified, such as *TTN*, *MUC4*,* KMT2C*, *SYNE1*, *DMD*, *USH2A*, *ADGRV1*, and *SYNE2* in CC,* TP53*, *TTN*, *CSMD3*, *MUC16*, *STNE1*, *FLG*, and *PCLO* in HNSCC (Figure 5A-D).

To identify the co-occurrence mutation signature between CC and HNSCC, 289 common co-occurrence mutation genes were merged (Figure 5E, Supplementary Table 5), which were enriched in some cancer pathways, such as proteoglycans in cancer (*P* = 3.55e-05), positive regulation of phosphatidylinositol 3-kinase (PI3K) signaling (*P* = 9.77e-05), microtubule-based movement (*P* = 4.79e-05), and chromosome segregation (P = 0.0002), et al. (Figure 5F). In particular, the protein-protein interaction network relating to chromosome segregation pathway was enriched (Figure 5G), genes (*ANK2*, *FN1*, *DYNC1H1*, *ANK3*, *CENPE*, *CENPF*, *PDS5B*, *BUB1*, *DDX18*, *MARS*, and *RAB1B*) were co-occurred in *LRP1B* mutation samples of CC and HNSCC (Figure 5H).

### Molecular characteristics of co-occurrence mutation signature of *LRP1B* in CC and HNSCC

**Figure 6:**
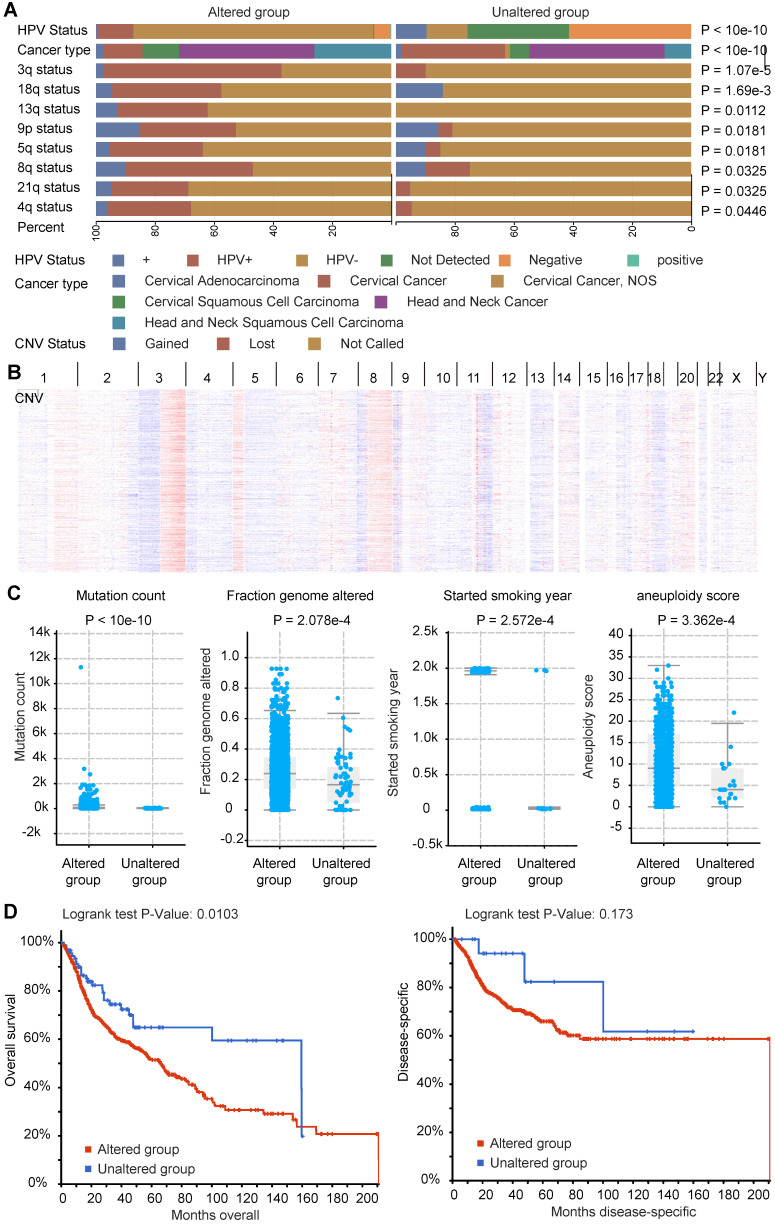
** Molecular characteristics of co-occurrence mutation signature of *LRP1B* in CC and HNSCC.** (**A**) CC and HNSCC samples were clustered into two groups according to *LRP1B* co-occurrence mutation signature status, different HPV status of samples, CNV status of the chromosome, cancer types were showed by color. Color captions were shown at the bottom. (**B**) CNV alteration of samples with the signature mutation was showed. (**C**) Mutation count, fraction genome altered (FGA), started smoking year, aneuploidy score of samples in groups according to 289 mutation genes status. (**D**) Survival analysis of samples in groups according to 289 mutation genes status. The altered group represents samples with at least one of 289 mutation genes corresponding to the co-occurrence mutation signature of *LRP1B*.

In accordance with the above co-occurrence mutation signature of *LRP1B* in CC and HNSCC, we performed molecular analysis of such 289 genes in cBioPortal tool, including HPV status, cancer types, CNV status, et al (Supplementary Table 6). By using *LRP1B* co-occurrence mutation signature, we identified the groups with different portion of HPV status (*P* < 10e-10), and different CNV status in genome. CNV lost as preferred in *LRP1B* co-occurrence mutation signature group, especially in 3q (*P* = 1.07e-5), 18q (*P* = 1.69e-3), 13q (*P* = 0.0112), 9p (*P* = 0.0181), 5q (*P* = 0.0181), 8q (*P* = 0.0325), 21q (*P* = 0.0325), and 4q (*P* = 0.0446) (Figure 6A), which were showed in Figure 6B.

Also, higher mutation count (*P* < 10e-10), higher altered fraction genome (*P* = 2.572e-4), and longer smoking year (*P* = 2.572e-4), and higher aneuploidy score (*P* = 3.362e-4) were found in *LRP1B* co-occurrence mutation signature group (Figure 6C). Furthermore, poor disease outcomes (overall survival, *P* = 0.0103) were found in *LRP1B* co-occurrence mutation signature group (Figure 6D).

## Discussion

Previous studies have demonstrated that HPV integration correlates with disease outcomes, host immune response signatures, cellular differentiation, dysregulation of the expression of viral E6 and E7 oncogenes, and aberrant expression of the integrated cancer-related genes in CC and HNSCC 33. While somatic mutations, such as *PIK3CA*, *PTEN*, *TP53*, *STK11*, and *KRAS*, and copy-number alterations in the pathogenesis of CC and HNSCC were significantly identified 2, 4, 22, 23. However, the correlation between HPV integration and gene mutation remains to be determined. As suggested by our results, *LRP1B* mutation was identified to be associated with tumor HPV status in CC and HNSCC and be associated with higher total E6 expression and spliced E6 expression in CC, which might be responsible for the correlation between genomic alteration accumulation and viral genome integration.

*LRP1B* belongs to the gene family of low-density lipoprotein (LDL) receptor, which plays multi-roles as a tumor suppressor gene in normal cell function and development 34. Down-regulation of *LRP1B* was reported to promote cell growth and migration in colon cancer cells 35, and in renal cell cancer 36, and is associated with acquired chemotherapy resistance in high-grade serous ovarian cancer 37. In accordance with our results that *LRP1B* mutation tended to down-regulated *LRP1B* expression and showed poor disease outcome in CC and HNSCC. In addition, *LRP1B* mutation serves as a good predictor for ICIs in multiple cancer types, largely due to the correlation with higher tumor mutation burden 12, 17. In CC and HNSCC, our results demonstrated that *LRP1B* mutation, as well as *LRP1B* co-occurrence mutation signature, were associated with higher mutation count. Higher tumor mutational burden shows distinct clinicopathologic features and is a biomarker for good efficacy and clinical response to ICIs in diverse cancers 38.

*LRP1B* was also detected as an HPV integration hotspot in CC 27, as suggested by our results, HPV-*LRP1B* integration was associated with the downregulation of *LRP1B*. Additionally, the LRP1B IHC staining score could serve as a sensitive biomarker for cervical lesion detection, with 0.801 area under curve (AUC), 90% sensitivity, and 56.76% specificity 39. In our results, the expression status of* LRP1B* was identified as a biomarker for normal and neoplasm samples of CC and HNSCC, with AUC 0.8007 in HNSCC and 0.9024 in CC, which suggested that *LRP1B* could serve as a biomarker in CC and HNSCC. Furthermore, *LRP1B* co-occurrence mutation signature tended to identify a cluster sample with multiple molecular bases of CC and HNSCC. The identification of molecular patterns or biomarkers of CC and HNSCC that could not only predict the tumor biology but help to further classify HPV-related cancers.

Still, with multi-molecular analyses in CC and HNSCC, our studies had several limitations in *LRP1B* mutation characteristics. Firstly, the correlation between *LRP1B* mutation and tumor HPV status was lack of molecular evidence and underlying mechanisms in the interaction process. Secondly, the biomarker role of *LRP1B* in ICIs was showed in other cancer types, there was no ICIs treatment cohort of CC or HNSCC included for clinical analysis, making the biomarker role of *LRP1B* only focus on cancer survival, mutation count, AUC analysis, and molecular bases of CC and HNSCC. The only way to demonstrate the molecular function of *LRP1B* was cell biology tests, which need to be further explored in HPV-related CC and HNSCC.

Overall, by integrating molecular data of CC and HNSCC, including gene mutation, tumor HPV status, gene expression, and clinical information, we identified *LRP1B* mutation, as well as co-occurrence mutation signature of *LRP1B*, was associated with tumor HPV status, higher mutation count, and poor cancer survival in CC and HNSCC. Also, *LRP1B* was showed as a good biomarker between normal and neoplasm samples than *TP53*, and *RB1* in CC and HNSCC. These results provided molecular insight into the correlation between HPV status and *LRP1B* mutation, and the prognostic value of *LRP1B* in HPV-related CC and HNSCC.

## Supplementary Material

Supplementary figures.

Supplementary tables.

## References

[1] Gillison ML, Chaturvedi AK, Lowy DR (2008). HPV prophylactic vaccines and the potential prevention of noncervical cancers in both men and women. Cancer.

[2] Ojesina AI, Lichtenstein L, Freeman SS, Pedamallu CS, Imaz-Rosshandler I, Pugh TJ (2014). Landscape of genomic alterations in cervical carcinomas. Nature.

[3] Chaturvedi AK, Engels EA, Pfeiffer RM, Hernandez BY, Xiao W, Kim E (2011). Human papillomavirus and rising oropharyngeal cancer incidence in the United States. J Clin Oncol.

[4] Cancer Genome Atlas N (2015). Comprehensive genomic characterization of head and neck squamous cell carcinomas. Nature.

[5] de Martel C, Plummer M, Vignat J, Franceschi S (2017). Worldwide burden of cancer attributable to HPV by site, country and HPV type. Int J Cancer.

[6] Winer RL, Hughes JP, Feng Q, Stern JE, Xi LF, Koutsky LA (2016). Incident Detection of High-Risk Human Papillomavirus Infections in a Cohort of High-Risk Women Aged 25-65 Years. J Infect Dis.

[7] Ho GY, Bierman R, Beardsley L, Chang CJ, Burk RD (1998). Natural history of cervicovaginal papillomavirus infection in young women. N Engl J Med.

[8] Rositch AF, Koshiol J, Hudgens MG, Razzaghi H, Backes DM, Pimenta JM (2013). Patterns of persistent genital human papillomavirus infection among women worldwide: a literature review and meta-analysis. Int J Cancer.

[9] Doorbar J, Quint W, Banks L, Bravo IG, Stoler M, Broker TR (2012). The biology and life-cycle of human papillomaviruses. Vaccine.

[10] Schiffman M, Doorbar J, Wentzensen N, de Sanjose S, Fakhry C, Monk BJ (2016). Carcinogenic human papillomavirus infection. Nat Rev Dis Primers.

[11] Hanahan D, Weinberg RA (2011). Hallmarks of cancer: the next generation. Cell.

[12] Zhu J, Tucker MD, Kao C, Labriola M, Cheris S, Datto MB Immune checkpoint inhibitor response in tumors with LRP1B variants. 2019; 37: e14291-e.

[13] Wang L, Yan K, Zhou J, Zhang N, Wang M, Song J Relationship of liver cancer with LRP1B or TP53 mutation and tumor mutation burden and survival. 2019; 37: 1573-.

[14] Kidd EA, Grigsby PW (2008). Intratumoral metabolic heterogeneity of cervical cancer. Clin Cancer Res.

[15] Leemans CR, Braakhuis BJ, Brakenhoff RH (2011). The molecular biology of head and neck cancer. Nat Rev Cancer.

[16] Bachtiary B, Boutros PC, Pintilie M, Shi W, Bastianutto C, Li JH (2006). Gene expression profiling in cervical cancer: an exploration of intratumor heterogeneity. Clin Cancer Res.

[17] Chen H, Chong W, Wu Q, Yao Y, Mao M, Wang X (2019). Association of LRP1B Mutation With Tumor Mutation Burden and Outcomes in Melanoma and Non-small Cell Lung Cancer Patients Treated With Immune Check-Point Blockades. Front Immunol.

[18] Tucker MD, Zhu J, Marin D, Gupta RT, Gupta S, Berry WR (2019). Pembrolizumab in men with heavily treated metastatic castrate-resistant prostate cancer. Cancer Med.

[19] Li J, Wei Q, Wu X, Sima J, Xu Q, Wu M (2020). Integrative clinical and molecular analysis of advanced biliary tract cancers on immune checkpoint blockade reveals potential markers of response. Clin Transl Med.

[20] Cao C, Lin S, Zhi W, Lazare C, Meng Y, Wu P (2020). LOXL2 Expression Status Is Correlated With Molecular Characterizations of Cervical Carcinoma and Associated With Poor Cancer Survival via Epithelial-Mesenchymal Transition (EMT) Phenotype. Front Oncol.

[21] Cao C, Hong P, Huang X, Lin D, Cao G, Wang L (2020). HPV-CCDC106 integration alters local chromosome architecture and hijacks an enhancer by three-dimensional genome structure remodeling in cervical cancer. J Genet Genomics.

[22] Cancer Genome Atlas Research N, Albert Einstein College of M, Analytical Biological S, Barretos Cancer H, Baylor College of M, Beckman Research Institute of City of H (2017). Integrated genomic and molecular characterization of cervical cancer. Nature.

[23] Stransky N, Egloff AM, Tward AD, Kostic AD, Cibulskis K, Sivachenko A (2011). The mutational landscape of head and neck squamous cell carcinoma. Science.

[24] Agrawal N, Frederick MJ, Pickering CR, Bettegowda C, Chang K, Li RJ (2011). Exome sequencing of head and neck squamous cell carcinoma reveals inactivating mutations in NOTCH1. Science.

[25] Pickering CR, Zhang J, Yoo SY, Bengtsson L, Moorthy S, Neskey DM (2013). Integrative genomic characterization of oral squamous cell carcinoma identifies frequent somatic drivers. Cancer Discov.

[26] Hoadley KA, Yau C, Hinoue T, Wolf DM, Lazar AJ, Drill E (2018). Cell-of-Origin Patterns Dominate the Molecular Classification of 10,000 Tumors from 33 Types of Cancer. Cell.

[27] Hu Z, Zhu D, Wang W, Li W, Jia W, Zeng X (2015). Genome-wide profiling of HPV integration in cervical cancer identifies clustered genomic hot spots and a potential microhomology-mediated integration mechanism. Nat Genet.

[28] Gerstung M, Pellagatti A, Malcovati L, Giagounidis A, Porta MG, Jadersten M (2015). Combining gene mutation with gene expression data improves outcome prediction in myelodysplastic syndromes. Nat Commun.

[29] Zhou Y, Zhou B, Pache L, Chang M, Khodabakhshi AH, Tanaseichuk O (2019). Metascape provides a biologist-oriented resource for the analysis of systems-level datasets. Nat Commun.

[30] Yang L, Yang H, Wu K, Shi X, Ma S, Sun Q (2014). Prevalence of HPV and variation of HPV 16/HPV 18 E6/E7 genes in cervical cancer in women in South West China. J Med Virol.

[31] Chalmers ZR, Connelly CF, Fabrizio D, Gay L, Ali SM, Ennis R (2017). Analysis of 100,000 human cancer genomes reveals the landscape of tumor mutational burden. Genome Med.

[32] Goodman AM, Kato S, Bazhenova L, Patel SP, Frampton GM, Miller V (2017). Tumor Mutational Burden as an Independent Predictor of Response to Immunotherapy in Diverse Cancers. Mol Cancer Ther.

[33] Koneva LA, Zhang Y, Virani S, Hall PB, McHugh JB, Chepeha DB (2018). HPV Integration in HNSCC Correlates with Survival Outcomes, Immune Response Signatures, and Candidate Drivers. Mol Cancer Res.

[34] Liu CX, Li Y, Obermoeller-McCormick LM, Schwartz AL, Bu G (2001). The putative tumor suppressor LRP1B, a novel member of the low density lipoprotein (LDL) receptor family, exhibits both overlapping and distinct properties with the LDL receptor-related protein. J Biol Chem.

[35] Wang Z, Sun P, Gao C, Chen J, Li J, Chen Z (2017). Down-regulation of LRP1B in colon cancer promoted the growth and migration of cancer cells. Exp Cell Res.

[36] Ni S, Hu J, Duan Y, Shi S, Li R, Wu H (2013). Down expression of LRP1B promotes cell migration via RhoA/Cdc42 pathway and actin cytoskeleton remodeling in renal cell cancer. Cancer Sci.

[37] Cowin PA, George J, Fereday S, Loehrer E, Van Loo P, Cullinane C (2012). LRP1B deletion in high-grade serous ovarian cancers is associated with acquired chemotherapy resistance to liposomal doxorubicin. Cancer Res.

[38] Yarchoan M, Hopkins A, Jaffee EM (2017). Tumor Mutational Burden and Response Rate to PD-1 Inhibition. N Engl J Med.

[39] Jiang Y, Zhu C, He D, Gao Q, Tian X, Ma X (2019). Cytological Immunostaining of HMGA2, LRP1B, and TP63 as Potential Biomarkers for Triaging Human Papillomavirus-Positive Women. Transl Oncol.

[40] Tang Z, Li C, Kang B, Gao G, Li C, Zhang Z (2017). GEPIA: a web server for cancer and normal gene expression profiling and interactive analyses. Nucleic Acids Res.

