# Supplementary material for: LRP1B mutation is associated with tumor HPV status and promotes poor disease outcomes with a higher mutation count in HPV-related cervical carcinoma and head & neck squamous cell carcinoma
Source: Int J Biol Sci. 2021 Apr 22;17(7):1744–56. doi: 10.7150/ijbs.56970 (PMC8120457; doi:10.7150/ijbs.56970)
Supplement: Supplementary file 1 — Supplementary figures. [file ijbsv17p1744s1.pdf]

Supplementary Figure 1: Mutation status in HPV A7 CC.

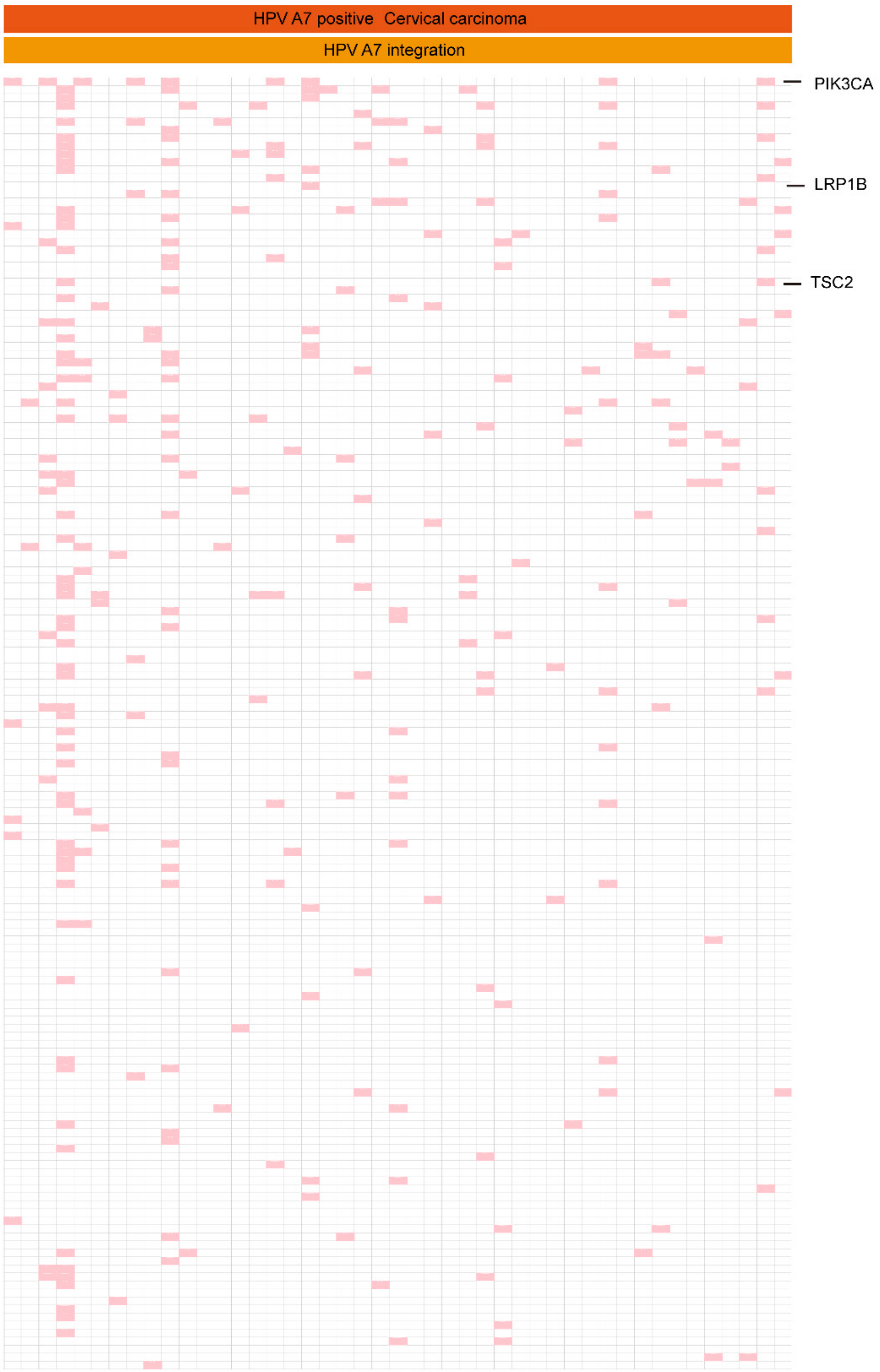

Supplementary Figure 2: Expression of *LRP1B* in cell lines with different *LRP1B* status. (**A**) Statistical analysis of immunofluorescence staining. (**B**) Mutation status of *LRP1B* in cell lines. *LRP1B* expreesion in cell lines from GSE124462 (**C**) and GSE39067 (**D**)

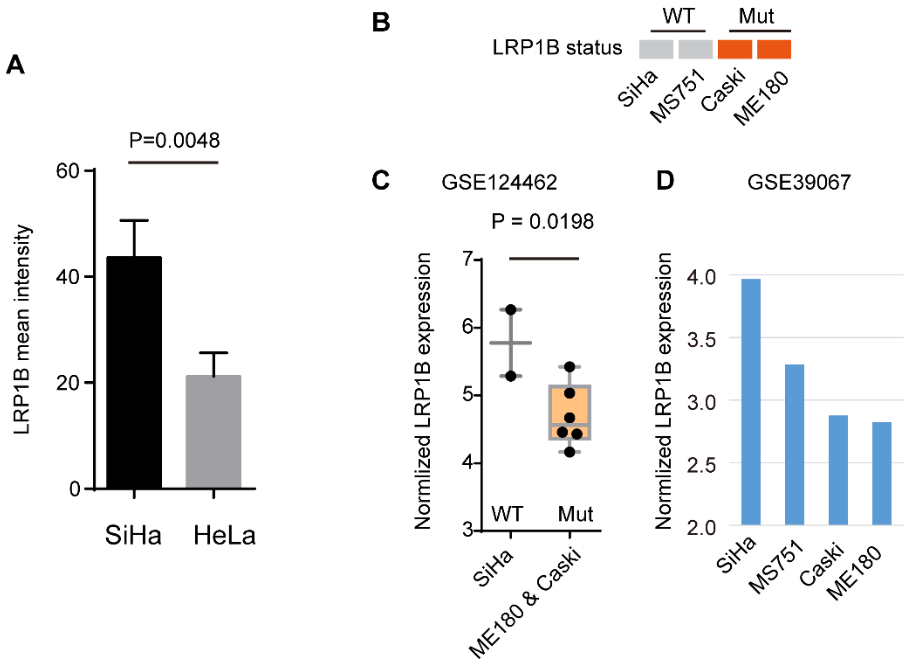

Supplementary Figure 3: Survival analysis in CC and HNSCC. Survival analysis in 33 cancer types (**A**), in CC (**B**), and HNSCC (**C**), all results were performed by GEPIA [40].

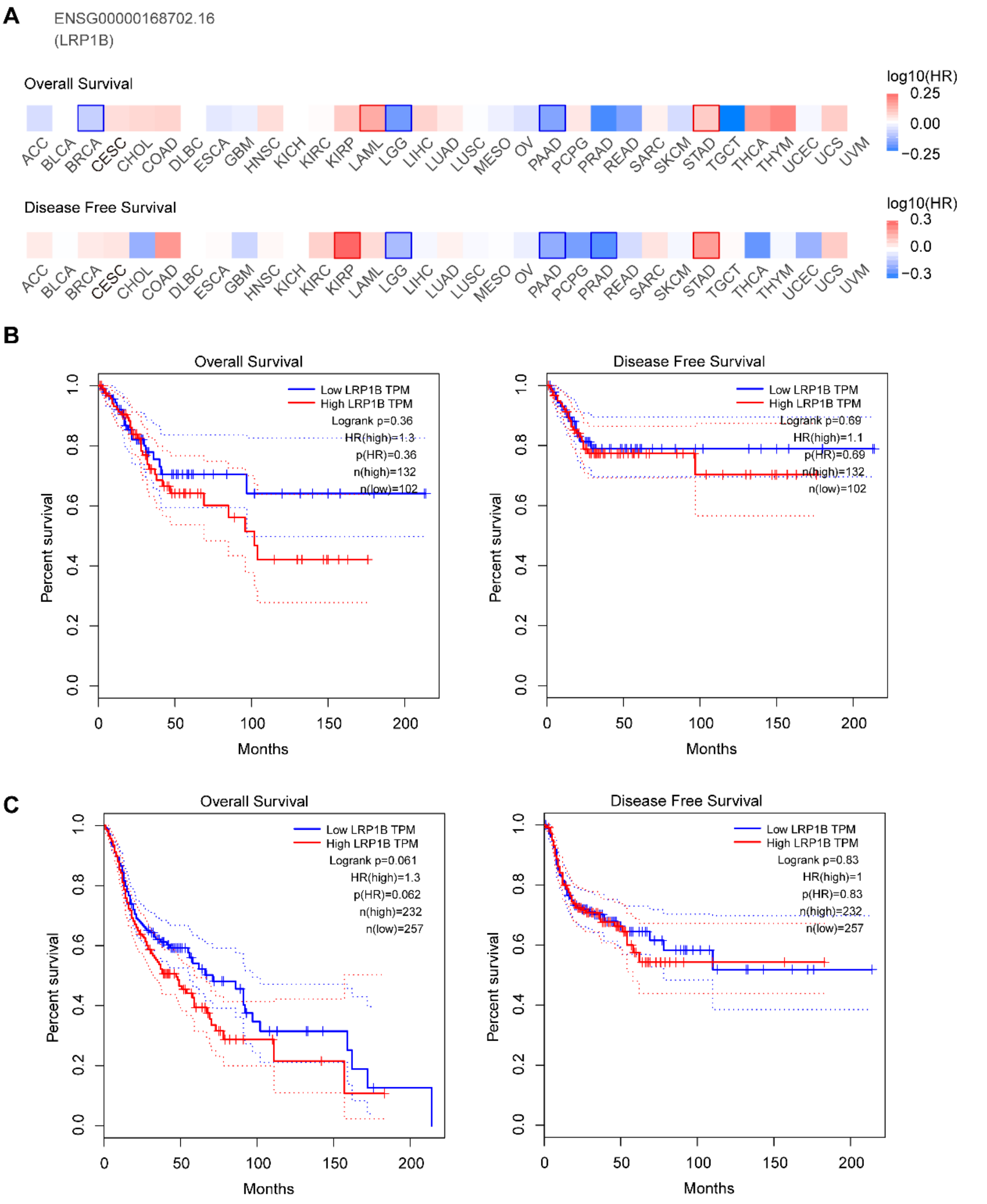

Supplementary Table 1: Mutation genes of HeLa cells.

Supplementary Table 2: Mutation genes of Caski cells.

Supplementary Table 3: Mutation genes of Me180 cells.

Supplementary Table 4: Samples information of CC cohort 1.

Supplementary Table 5: Description of 289 genes.

Supplementary Table 6: Molecular analyses of 289 co-occurrence mutation genes.
